# Comparative Thrombin Generation in Animal Plasma: Sensitivity to Human Factor XIa and Tissue Factor

**DOI:** 10.3390/ijms241612920

**Published:** 2023-08-18

**Authors:** Yideng Liang, Ivan Tarandovskiy, Stepan S. Surov, Mikhail V. Ovanesov

**Affiliations:** Office of Tissues and Advanced Therapies, Center for Biologics Evaluation and Research, Food and Drug Administration, Silver Spring, MD 20993, USA; yideng.liang@fda.hhs.gov (Y.L.); ivan.tarandovskiy@fda.hhs.gov (I.T.); stepan.surov@fda.hhs.gov (S.S.S.)

**Keywords:** thrombin generation, fibrin clot formation, animal model, tissue factor, activated factor XI, assay sensitivity

## Abstract

Preclinical evaluation of drugs in animals helps researchers to select potentially informative clinical laboratory markers for human trials. To assess the utility of animal thrombin generation (TG) assay, we studied the sensitivity of animal plasmas to triggers of TG, human Tissue Factor (TF), and Activated Factor XI (FXIa). Pooled human, mouse, rat, guinea pig, rabbit, bovine, sheep, and goat plasmas were used in this study. TF- or FXIa-triggered TG and clotting were measured via fluorescence and optical density, respectively. Thrombin peak height (TPH) and time (TPT), clot time (CT), and fibrin clot density (FCD) were all analyzed. The trigger low and high sensitivity borders (LSB and HSB) for each assay parameter were defined as TF and FXIa concentrations, providing 20 and 80% of the maximal parameter value, unless the baseline (no trigger) value exceeded 20% of the maximal, in which case, LSB was derived from 120% of baseline value. Normal human samples demonstrated lower TPH HSB than most of the animal samples for both TF and FXIa. Animal samples, except mice, demonstrated lower TPT LSB for FXIa versus humans. Most rodent and rabbit samples produced baseline TG in the absence of TG triggers that were consistent with the pre-activation of blood coagulation. FCD was not sensitive to both TF and FXIa in either of the plasmas. Animal plasmas have widely variable sensitivities to human TF and FXIa, which suggests that optimization of trigger concentration is required prior to test use, and this complicates the extrapolation of animal model results to humans.

## 1. Introduction

Thrombin generation (TG) assay is a popular research tool that is used to evaluate the hemostasis potential of both anti- and pro-coagulant drugs in human patients and animal models of the disease [1,2]. The most common TG approach is measuring fluorescence produced by 7-amino-4-methylcoumarin that is released when thrombin cleaves a specific peptide substrate added to plasma. The assay is usually triggered via extrinsic pathway to mimic physiological coagulation activation by tissue factor (TF) that is exposed at the site of the injured endothelium [3]. Activated coagulation factor XI (FXIa), which is a product of the contact activation, can be used together with or instead of TF in order to stimulate the intrinsic pathway of the coagulation cascade, e.g., to measure the treatment of severe deficiencies of coagulation factors VIII and IX (hemophilia A and B) [4,5,6]. Compared to traditional functional hemostasis assays, such as Prothrombin Time (PT) and activated Partial Thromboplastic Time (aPTT), TG may show better sensitivity to both disease conditions and the effects of novel treatments. For this reason, TG is often used in the preclinical evaluation of diseases and drugs in animal models.

Extrapolation of animal study results to humans is affected by interspecies differences in the scale and function of the blood circulatory system as well as physiology and biochemistry of the hemostasis system [7,8,9,10]. The practical usefulness of an animal model depends on the similarity of the recorded coagulation responses in animals to those in humans, and, therefore, justification is needed for choosing the most suitable species for a particular scientific study. TG assay is sensitive to coagulation factor concentrations and activities, both of which are known to vary across different species [11,12,13]. Preanalytical conditions also strongly influence TG [7,14]. Despite this, TG is widely used for evaluation of coagulation in animal models, often without a side-by-side comparison between the animal and the human plasma samples under study. Moreover, there is a limited amount of studies that compare TG between various species [8,10,15,16]. When interspecies differences were evaluated [9,17,18,19], the authors suggested that while some animals might have TG similar to humans, others might not. This type of data can bring useful evidence forward for coagulation investigations that are species specific. Importantly, no study to-date has compared the TG in different animal species over a wide range of coagulation trigger concentrations. Indeed, species variations in coagulation factor activity can lead to inconsistent sensitivity of the coagulation cascade to both the extrinsic and intrinsic activation pathways in different animal samples [20]. For animal studies to be potentially informative of human trials, biology and assays in animals need to be relevant for clinical laboratory methods in humans.

**Figure 1:**
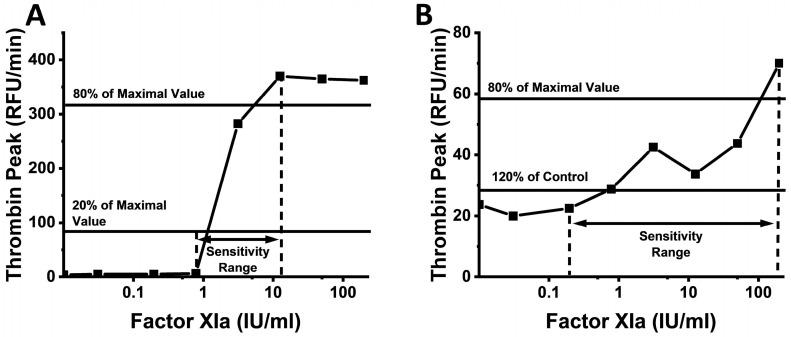
TF and FXIa sensitivity ranges for each parameter were defined as higher and lower sensitivity boundaries, respectively. Representative dependencies of TPH on FXIa concentration in human (**A**) and guinea pig (**B**) samples.

In the present study, to assess the utility of animal thrombin generation (TG) assay, we studied the sensitivity of animal plasmas to triggers of TG, human Tissue Factor (TF), and activated Factor XI (FXIa). There are no prior investigations of this kind, despite the knowledge that coagulation triggers can bring about a significant change in the effects of coagulation agents used in clinics and research after transferring them from animal to human samples. In the present study, we investigated the TG and fibrin clot formation (CF) sensitivity of various human and animal plasma samples to the main actors of the extrinsic and intrinsic pathways: TF and FXIa. We defined low and high sensitivity borders for each calculated parameter (Figure 1).

## 2. Results

**Figure 2:**
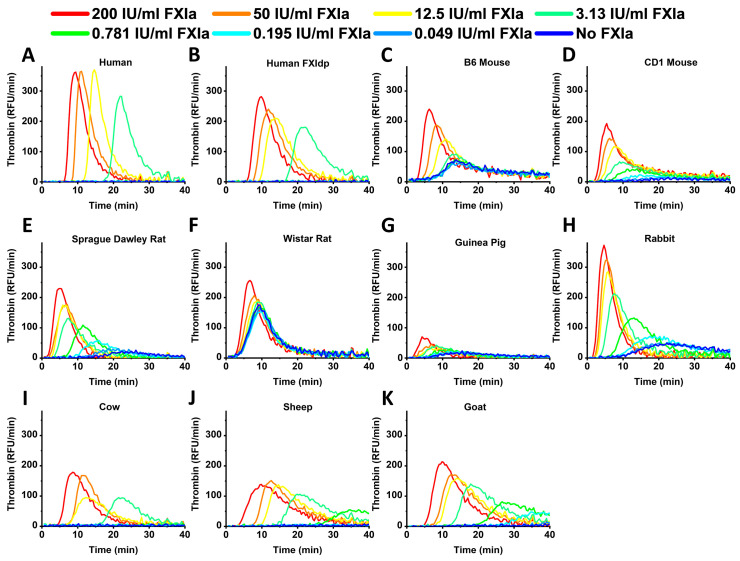
Representative TG curves obtained in normal human (**A**), human factor XI depleted (**B**), B6 mouse (**C**), CD1 mouse (**D**), Sprague Dawley rat (**E**), Wistar rat (**F**), guinea pig (**G**), rabbit (**H**), cow (**I**), sheep (**J**) and goat (**K**) plasma at different FXIa concentrations. Human and rabbit samples demonstrated the highest TPH values, while rodent samples showed lower TPT compared to human and cattle ones. Rodent and rabbit samples demonstrated detectable TG in blank samples without FXIa. Guinea pig plasma showed the lowest signal range.

Figure 2 represents the TG curves obtained in human and animal samples in the presence of different FXIa concentrations. Human and rabbit samples demonstrated the highest TPH values, while rodent samples showed the lowest TPT compared to human and cattle ones. Rodent and rabbit samples demonstrated detectable TG without FXIa presence. Guinea pig plasma showed the lowest signal range.

**Figure 3:**
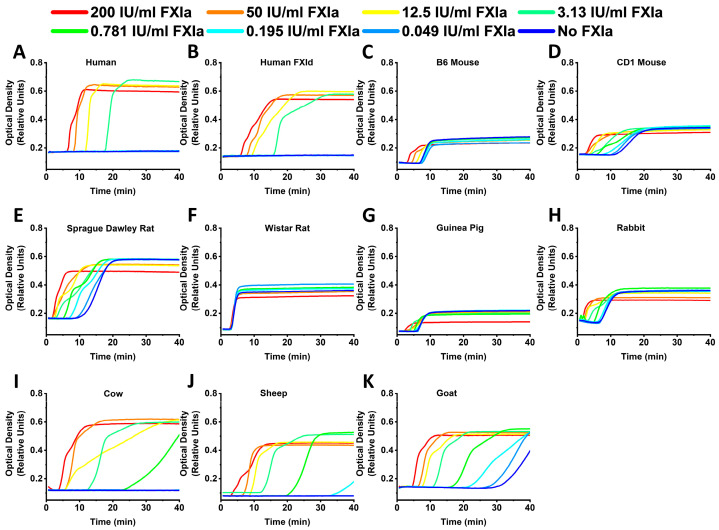
Representative CF curves obtained in normal human (**A**), human factor XI depleted (**B**), B6 mouse (**C**), CD1 mouse (**D**), Sprague Dawley rat (**E**), Wistar rat (**F**), guinea pig (**G**), rabbit (**H**), cow (**I**), sheep (**J**) and goat (**K**) plasma at different FXIa concentrations. All samples, except Wistar rats, demonstrated the dependence of CT on FXIa. For the samples with detectable CF, FCD was lower at higher FXIa. B6 and CD1 mice and guinea pigs showed the lowest FCD.

All of the animal samples, except the Wistar rats, demonstrated the dependence of CT on FXIa (Figure 3). In most of the samples with detectable CF, FCD showed a decay upon the elevation of FXIa concentration. FCD values obtained in both B6 and CD1 mice and guinea pigs were the lowest in the study.

**Figure 4:**
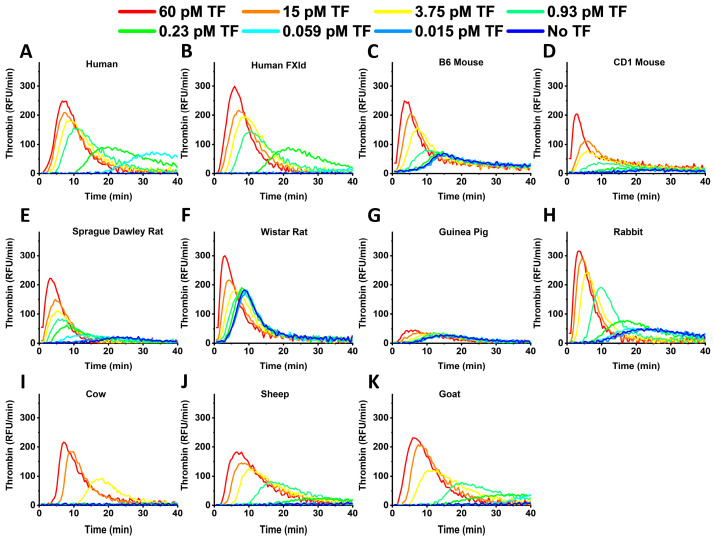
Representative TG curves obtained in normal human (**A**), human factor XI depleted (**B**), B6 mouse (**C**), CD1 mouse (**D**), Sprague Dawley rat (**E**), Wistar rat (**F**), guinea pig (**G**), rabbit (**H**), cow (**I**), sheep (**J**) and goat (**K**) plasma at different TF concentrations. Human and rabbit samples had the highest TPH values. Rodent samples had lower TPT values compared to human and cattle samples.

Analogously to FXIa-dependent TG, human and rabbit samples in the case of TF activation had the highest TPH values (Figure 4). Rodent samples had lower TPT values compared to human and cattle ones. In rodent samples, except guinea pigs, the initial thrombin values could not be accurately measured at high TF concentrations because of the small lag times, i.e., the peak of TG was happening too fast for our instruments, which were recording intervals of about 30 s.

**Figure 5:**
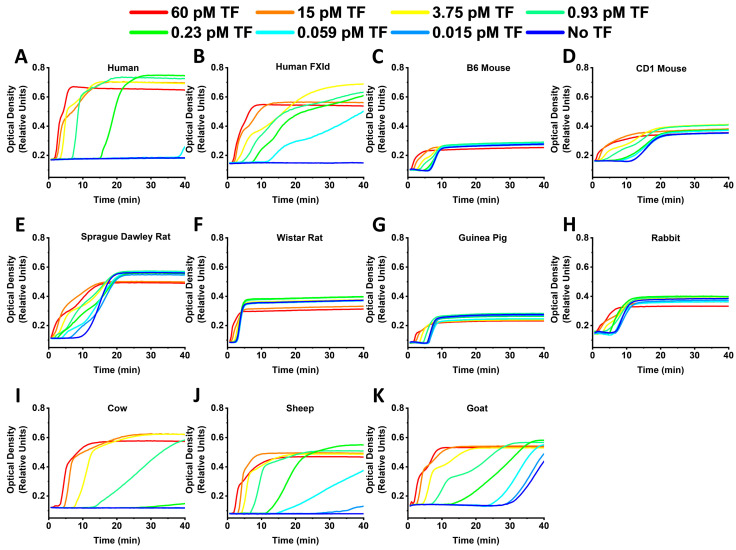
Representative CF curves obtained in normal human (**A**), human factor XI depleted (**B**), B6 mouse (**C**), CD1 mouse (**D**), Sprague Dawley rat (**E**), Wistar rat (**F**), guinea pig (**G**), rabbit (**H**), cow (**I**), sheep (**J**) and goat (**K**) plasma at different TF concentrations. CT was dependent on TF concentration in all samples. For samples with detectable CF, FCD was lower at higher TF.

CT was dependent on TF concentrations in all of the samples (Figure 5). Similar to FXIa, in the samples with detectable CF, FCD was decreasing following TF concentration elevation.

**Figure 6:**
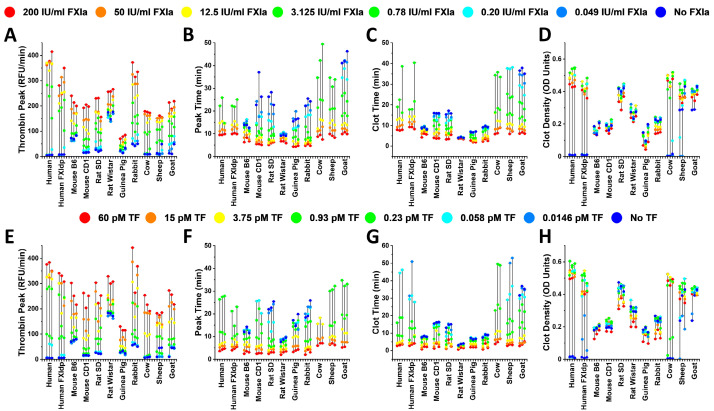
Ranges of responses measured by TG and CF parameters at different concentrations of FXIa (**A**–**D**) and TF (**E**–**H**). Colors of dots denote concentrations of TF and FXIa. Vertical lines show the response range from LSB to HSB. For each sample, the results of three independent experiments are shown.

Figure 6 presents the response range and value distributions of observed TG and CF parameters for all of the study samples. The human FXI deficient plasma sample demonstrated similarity to animals in the effects of TG and FXIa concentrations. Despite being originally from the same species, different lines of rats and mice were not similar to each other. Bovine, sheep, and goat samples were closer to one another than to humans, rodents, or rabbits.

**Figure 7:**
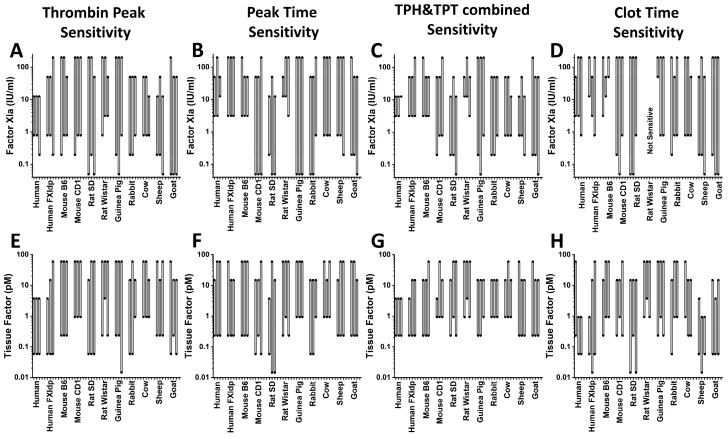
Ranges of FXIa (**A**–**D**) and TF (**E**–**H**) concentrations resolved by different TG and CT parameters are represented by grey boxes. For each sample, the results of three independent experiments are shown.

Figure 7 demonstrates the FXIa (Figure 7A–D) and TF (Figure 7E–H) ranges of sensitivity of TG and CF parameters. The LSB and HSB values are also shown in Table S1. FCD did not show any sensitivity to TF and FXIa. Normal human plasma had the narrowest FXIa sensitivity range of all of the tested plasmas. The TPH HSB of this sample had the lowest value: only one experiment on a cow sample and two of sheep ones demonstrated the same TPH HSB (Figure 7A). SD rat and goat samples had the widest TPH sensitivity range of FXIa concentrations (Figure 7A). TPT LSB values obtained in all of the animal samples, except the B6 mouse and Wistar rat ones, were lower than the ones from the normal human sample (Figure 7B). CD1 mouse and guinea pig had the widest TPH sensitivity range of the FXIa concentrations (Figure 7B). Combined TPH and TPT FXIa sensitivity ranges in the normal human plasma were the narrowest of all of the tested plasmas (Figure 7C). Guinea pig and goat samples had the widest range of combined TG sensitivity to FXIa. CT sensitivity ranges were similar to TPT sensitivity ranges, except for the Wistar rat samples, which did not show any CT sensitivity (Figure 7D). All of the samples had higher TPH TF HSB than the normal human one (Figure 7E). Both mice lines, Wistar rat, bovine, and lamb plasmas demonstrated higher TPH TF LSB than the normal human one (Figure 7E). TPT and combined TG sensitivity was more similar between all of the samples in the TF-dependent TG than in the FXIa-dependent one (Figure 7F,G). CT TF sensitivity varied widely from species to species (Figure 7H). Interestingly, human FXI deficient plasma showed wider sensitivity ranges not only to FXIa but to TF as well.

**Figure 8:**
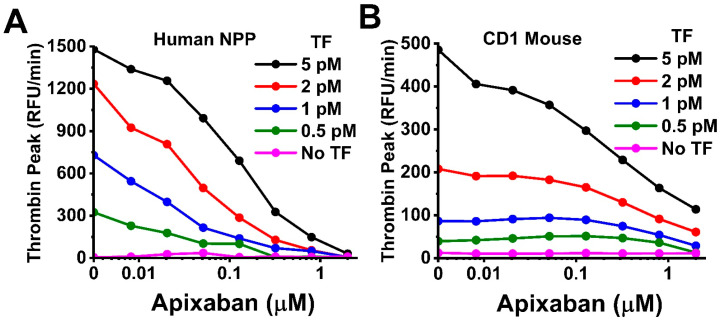
TPH dependencies on apixaban concentrations in the presence of 0, 0.5, 1, 2, and 5 pM TF in human (**A**) and CD1 mouse (**B**) samples.

To demonstrate the importance of inconsistency in sensitivity to a coagulation trigger between different species, TG was measured in a wide range of concentrations of direct factor Xa inhibitor apixaban in human and CD1 mouse samples in the presence of 0, 0.5, 1, 2, and 2 pM TF (Figure 8). According to Figure 7E, 1 pM is the LSB of TF sensitivity, and 0.5 pM of TF is below the LSB of CD1 mouse plasma, but is still well within the LSB to HSB range of the human sample. In the human sample, apixaban inhibited TG at all of the TF concentrations (Figure 8A). In CD1 mouse plasma, apixaban did not influence the TPH value at the concentrations below 1 µM in the presence of 0.5, 1, and 2 pM TF (Figure 8B).

## 3. Discussion

In this work, we have expanded our previous observations of the differences between human, bovine, and mouse plasma responses to human FXIa [18]. Preclinical evaluation of drugs in animals can assist in selecting informative laboratory methods for human trials, and it can help with dose extrapolation between species. In addition, animal models can be required to gain understanding of drug and disease mechanisms and effects when such information cannot be obtained in humans. For example, when direct and well-controlled observations of drug-mediated hemostasis in humans are prohibited due to technical or ethical concerns, research questions should be resolved in appropriate animal experiments. The predictive value of animal data for humans should consider the extent of differences in human and animal physiology. To assist with the selection of species for studies of FXIa, we systematically assessed the utility of animal TG assay by studying the sensitivity of animal plasmas to triggers of TG, human TF, and FXIa, and we used pooled human, mouse, rat, guinea pig, rabbit, bovine, sheep, and goat plasmas. TF- or FXIa-triggered TG and clotting were measured via fluorescence and optical density, respectively. We mapped a widely variable sensitivity to human TF and FXIa, suggesting that the optimization of trigger concentration will be required prior to both test use and the extrapolation of any results in animals to humans.

Because we aimed to develop models for studies of drugs that target human TF and FXIa, our study included only human TF and FXIa. Our results are relevant to previous investigations of TG in animals because global hemostasis assays traditionally rely on human TF to trigger coagulation in animal plasma. However, the structural differences between human and animal plasma proteins can bring additional inconsistency in the extrapolation of TG initiated by human triggers in animal plasma. Further investigation of animal species and coagulation triggers of corresponding sources can yield more information on animal models of TG.

Our analysis of animal species was designed to limit the variability of individual animal donors. We used pooled plasma to model the average population response, but we lacked information about possible subject-to-subject variability in TG sensitivity, which will need to be obtained in future studies that utilize single donor samples for each species. Our TG results appear consistent with previous studies of individual animal donors [8,9,10,15,16,17,18,19]. Consistent with previous reports [9,17,18], we observed that rat, mouse, and rabbit samples had smaller thrombin signals and lower TPT and clot times than human plasmas. TPH values obtained in sheep and goat plasma in our experiments and previously [9,15] were lower than in human plasma. Similar to others [18], we observed surprisingly low TG in guinea pigs. In agreement with previous work [19], too, we observed high TPH values in SD rat plasma in the absence of coagulation triggers, and this may be caused by the high procoagulant activity of rat plasma or the blood collection artifacts, including trace amounts of TF, contact system activation, and FXI activation. While we used CTI to block the contact enzyme FXIIa during the TG reaction, CTI does not inhibit FIXa produced by FXIIa or kallikrein during blood collection in the absence of CTI [21,22]. Interestingly, some previous studies disagreed on the presence of TG in CTI-treated mouse plasma without triggers [23,24]. We think that inconsistent TG results may be explained by differences in the blood collection methods or the animal lines. These concerns are not unique to our experiments, but they do represent another limitation for the extrapolation of TG results from rodents to humans.

Overall, our results obtained with the TF trigger both confirm and expand the previous observations about TG and CF in various species. Our investigation of the FXIa trigger is the first TG study on animal plasma. FXIa is a crucial component of the intrinsic pathway of blood coagulation activation [25]. FXIa and the intrinsic pathway have a limited role in hemostasis, but they are suspected to be contributors to thrombosis because FXI activation in blood has been demonstrated in both thromboembolic and inflammatory events [26,27,28,29,30,31,32]. Indeed, several pharmacological inhibitors that target FXIa are being developed for prevention of thrombosis with the hope of achieving anticoagulation with a reduced risk of bleeding [33,34]. Our study may assist in the preclinical evaluation of these inhibitors in animal species. To our knowledge, only two studies have previously observed TG triggered by FXIa in the plasma of animals, and both were performed in mice [16,35]. In the present study, we obtained results analogous to the study [16] in murine plasma, and we added a comparison to other species.

Our results suggest large interspecies inconsistency in TG sensitivity to TF and FXIa. The FXIa concentration threshold for TG response differed by orders of magnitude. Species can thus have an impact on the observations of coagulation agent actions, as seen in our apixaban titration experiments (Figure 8). In order to obtain meaningfully similar functional results in different animal species, one can choose drug doses using the ranges of sensitivity to coagulation triggers in plasmas from all animals that are to be used in a study, and one can then choose trigger concentrations within the LSB to HSB range for each sample. Figure 8 shows that choosing the TF concentration between the LSB and HSB for both the human and CD1 mouse results in measurable apixaban effect in both of the samples.

Interestingly, rodent samples demonstrated detectable TG even without addition of FXIa or TF. This could be due to the elevated activity of the contact pathway, which results in faster FIXa generation, as has been shown previously [6]. The preexisting hyperactivity of the contact and the intrinsic pathways may interfere with the procoagulant action of low concentrations of TF or FXIa, causing the low sensitivity that was observed in rodents. This low sensitivity mechanism can be indirectly supported by previous investigations of heparin activity in rats [19] and of TFPI activity in mice [36]. In the first study [19], in addition to low heparin sensitivity, TF concentration in a rat sample had limited effect. Similarly, the addition of TFPI to the plasma of mice that were deficient in TFPI [36] found no effect on TG triggered by TF, possibly due to the TF concentration of 0.1 pM being below the LSB in the mouse plasma that was observed in our study.

Our study has technical limitations that prevented us from calibrating thrombin activity or determining some TG curve parameters that are commonly used. These limitations are inherent to a study that uses many animal species, and they do not affect the main findings, which focus on the relative analyses of TG assay sensitivity to FXIa and TF, but they may complicate the quantitative comparison of TG data with other published works. For example, we did not perform the calculation of fluorescence rate to nanomoles of thrombin as this is conventionally done [1] due to the lack of information on the kinetics of animal thrombin-mediated conversion of fluorogenic substrate. In addition, the correct calculation of thrombin activity requires calibrators made of thrombin-α_2_macroglobulin complex, which are two proteins that are not currently available commercially for every species that we tested. In our study, we did not investigate endogenous thrombin potential (ETP) because this parameter strongly depends on the amount of thrombin bound to α2macroglobulin after the end of TG. At the same time, TPH usually correlates well with ETP. Similarly, the complex shape of TG curves in the plasma of some animals prevented us from accurately determining the lag time, although we assume that the time to thrombin peak (TPT) serves as a good surrogate for the lag time parameter.

In conclusion, our results are the first to demonstrate interspecies sensitivity to concentrations of TF and FXIa. We showed that TF and FXIa sensitivity can vary widely in different species. This can lead to difficulties in interpretation of drug action studies in animal models and humans. Therefore, we suggest that future animal model TG studies should include preliminary investigation of sensitivities to coagulation triggers rather than using off-the-shelf trigger reagents from the kits that are intended for human plasma.

## 4. Materials and Methods

Affinity-depleted Factor XI deficient plasma (FXI-DP) was from Affinity Biologicals (Ancaster, ON, Canada). The fluorogenic substrate, Z-Gly-Gly-Arg-AMC, was from Bachem Americas (King of Prussia, PA, USA). Phospholipid vesicles for TG assays was from Rossix (Molndal, Sweden). Lipidated human recombinant tissue factor (TF, Recombiplastin^®^) was from Instrumentation Laboratory (Bedford, MA, USA). Corn trypsin inhibitor (CTI) was from Haematologic Technologies (Essex Junction, VT, USA). CaCl_2_ was from Quality Biologicals (Gaithersburg, MD, USA). The World Health Organization (WHO) Reference Reagent for FXIa (coded 11/236) was from National Institute for Biological Standards and Control (NIBSC, London, UK). Pooled animal plasma samples from New Zealand white rabbits, Sprague Dawley (SD) rats, Wistar rats, sheep, and goats were from Innovative Research (Peary Court Novi, MI, USA), and guinea pig and bovine plasma was from Lampire Biological Laboratories (Pipersville, PA, USA). The 3.2% buffered sodium citrate was from Fisher Scientific, Waltham, MA, USA. The 3 mL syringes and 27 G x ⅜ hypodermic needles were from Beckton Dickinson, Franklin Lakes, NJ, USA). Crl:CD-1(ICR)BR (CD1) mice were from Charles River Laboratories International (Frederick, MD, USA), and C57BL6 (B6) mice were from Jackson Laboratory (Bar Harbor, ME, USA).

The terminal blood collection method was used for mouse plasma collection. A cardiac puncture was used, since it enables blood draws of up to 1 mL and the subsequent isolation of large amounts of plasma. Animals were anesthetized with a mouse cocktail IP injection (ketamine/xylazine, 0.1 mL/20 mg) and tested for reaction by toe pinch. After that, a mouse was placed on its back. The stomach was cut through the skin and abdominal wall about 1 cm caudal to the last rib, the internal organs were moved to the side, and a 3 mL syringe with the 27 g needle prefilled with 50 μL of 3.2% citrate buffer was inserted through the right heart. Whole blood was collected into a scaled 1.5 mL tube, and an additional 3.2% citrate buffer was added if needed to ensure a 1:9 ratio of citrate to blood. After that, whole blood was centrifuged at 2000 rpm for 15 min. The collected supernatant was centrifuged at 14,000 rpm for 10 min to prepare platelet poor plasma. To make pooled B6 and CD1 plasma, 20 animals of each line were used.

The animal protocol and procedures were approved by the FDA Institutional Animal Care and Use Committee (AUCUC), and all methods were performed according to relevant approved protocol.

TG was performed as described previously [37], but with slight modifications. Plasma (50% *vol*/*vol* in the final reaction) were set up in wells of a conical well microplate followed by the addition of a mixture of phospholipids (4 μM final concentration), corn trypsin inhibitor (CTI) (100 μg/mL final concentration), TF, or FXIa (0.015 to 60 pM and 0.049 to 200 IU/mL, respectively), and the remaining volume was Tris-BSA buffer (pH 7.4, Aniara, West Chester, OH, USA). Clotting was initiated by transferring the plasma mixture into a half-area 96-well flat bottom plate containing CaCl_2_ (10.9 mM final concentration) and fluorogenic substrate Z-Gly-Gly-Arg-AMC (1.25% *vol*/*vol*, 800 μM final concentration) using a 96-channel pipettor (ViaFlo 96, Integra Biosciences, Hudson, NH, USA) in order to permit rapid and simultaneous recalcification in all of the wells of the microplate. The flat bottom plate, which contained the activated reaction mixture, was transferred to an Infinite F500 (Tecan, Männedorf, Switzerland) microplate reader regulated at 37⁰ C. Fluoresence (380 nm excitation, 430 nm emission) and absorbance (492 nm) were recorded every 40–50 s.

TF and FXIa sensitivity ranges for each parameter were defined as the higher and lower sensitivity borders (LSB and HSB, respectively; Figure 1). For the samples with poor or undetectable TG or clotting signal in the control sample (plasma sample without added TF or FXIa), the LSB and HSB were defined as the TF or FXIa concentrations providing 20% (for LSB) and 80% (for HSB) of the maximal parameter value, respectively, for each experiment (Figure 1A). For the plasma samples in which the blank responses (i.e., TG without TF or FXIa triggers) were higher than 1/5th (20%) of the maximal responses, 120% of the blank value was used as the LSB (Figure 1B). In order to obtain the overall TG sensitivity over all of the TG parameters combined, the highest LSB and the lowest HSB values were selected by comparing the LSB and HSB values for TPH with those for TPT.

Y. Liang, I. Tarandovskiy, S. S. Surov, and M.V. Ovanesov are employees of the U.S. Food and Drug Administration. This is an informal communication, and it represents the own best judgment of the authors. These comments do not bind or obligate the FDA.

## Data Availability

Data supporting reported results can be available upon request.

## Supplementary Materials

The following supporting information can be downloaded at: https://www.mdpi.com/article/10.3390/ijms241612920/s1.
